# Management of Infection by Parasitic Weeds: A Review

**DOI:** 10.3390/plants9091184

**Published:** 2020-09-11

**Authors:** Mónica Fernández-Aparicio, Philippe Delavault, Michael P. Timko

**Affiliations:** 1 Institute for Sustainable Agriculture, Consejo Superior de Investigaciones Científicas (CSIC), 14004 Córdoba, Spain; 2 Laboratory of Plant Biology and Pathology, University of Nantes, 44035 Nantes, France; Philippe.Delavault@univ-nantes.fr; 3 Department of Biology University of Virginia, Charlottesville, VA 22904-4328, USA; mpt9g@virginia.edu

**Keywords:** *Orobanche*, *Phelipanche*, *Striga*, *Cuscuta*, germination, haustorium, crop resistance, bioherbicides, virulence, sustainable control

## Abstract

Parasitic plants rely on neighboring host plants to complete their life cycle, forming vascular connections through which they withdraw needed nutritive resources. In natural ecosystems, parasitic plants form one component of the plant community and parasitism contributes to overall community balance. In contrast, when parasitic plants become established in low biodiversified agroecosystems, their persistence causes tremendous yield losses rendering agricultural lands uncultivable. The control of parasitic weeds is challenging because there are few sources of crop resistance and it is difficult to apply controlling methods selective enough to kill the weeds without damaging the crop to which they are physically and biochemically attached. The management of parasitic weeds is also hindered by their high fecundity, dispersal efficiency, persistent seedbank, and rapid responses to changes in agricultural practices, which allow them to adapt to new hosts and manifest increased aggressiveness against new resistant cultivars. New understanding of the physiological and molecular mechanisms behind the processes of germination and haustorium development, and behind the crop resistant response, in addition to the discovery of new targets for herbicides and bioherbicides will guide researchers on the design of modern agricultural strategies for more effective, durable, and health compatible parasitic weed control.

## 1. Introduction

**Figure 1:**
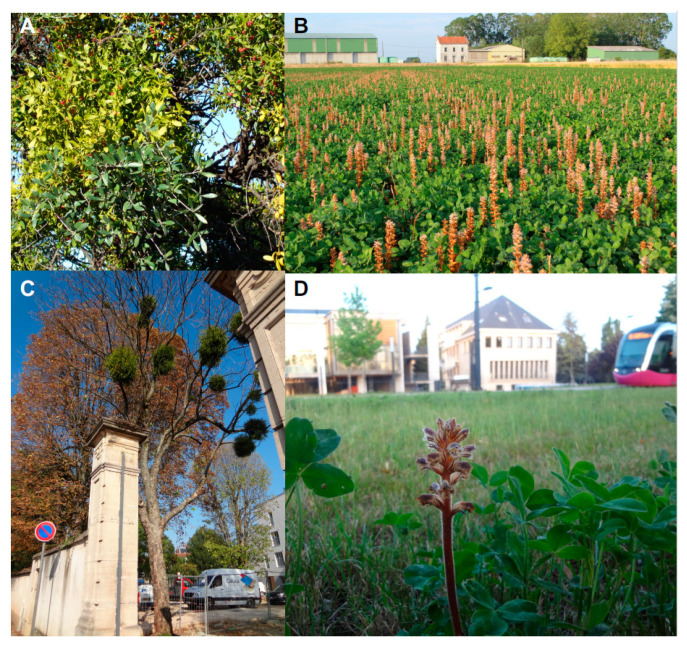
Adaptation of parasitic plants to agricultural and urban ecosystems. Shown are representative photographs of parasitic plants in agricultural (**A**,**B**) and urban (**C**,**D**) locations. (**A**) Hemiparasitic shoot parasite *Viscum* sp. feeding on olive tree; (**B**) holoparasitic root parasite *Orobanche* sp. feeding on clover commercial field; (**C**) *Viscum* sp., in the city of Dijon, France; (**D**) *Orobanche* sp. parasitizing clover in a park in the city of Dijon, France.

Approximately 1% of all angiosperms are parasitic on other plants and these plants are distributed among 28 dicotyledonous families having evolved the parasitic lifestyle independently at least 12 times [1,2,3,4,5]. Some are facultative parasites, capable of living autotrophically until reproduction, but shifting to a parasitic life form when a host is available to obtain nutrients with less investment in assimilation system. In comparison, obligated parasitic plants require the infection of another plant to survive shortly after germination. Parasitic plants can be also grouped by their photosynthesis competency, being either photosynthetically active hemiparasites or achlorophyllous holoparasites; or separated based upon the type of vascular connections they form with their host, being either xylem feeders or phloem feeders. They can also be grouped by the host plant organ to which they attach, either root feeders or shoot feeders [4,6]. While many parasitic plants have remained part of larger ecological communities, a small number of species have evolved to weediness, becoming troublesome pests in the agricultural field and an important constraint to crop productivity. Besides colonizing agroecosystems, parasitic plants are also present in urban ecosystems (Figure 1). 

While all agricultural weeds compete with crops for the space to obtain water, nutrients, and light, parasitic weeds are particularly noxious since they also directly extract valuable water and nutrients from the host plant. To extract nutrients from the host plants, parasitic weeds have evolved a unique multicellular structure termed the haustorium that invades the host, forms connections with the host vascular system, and withdraws its needed water and nutrients [7,8]. Successful haustorial connection to the host results in permanent damage during a large part of the crop life cycle, decreasing the crop value by reducing the harvested yield and contaminating it with parasitic seeds. Worldwide expansion of these noxious plant pests including the widely recognized genera *Striga*, *Orobanche/Phelipanche*, and *Cuscuta* has become a threat to food security [9].

There are few sources of crop resistance to parasitic plant infection [10,11]. The identified forms of resistance are classified as pre-attachment or post-attachment resistance according to whether the resistance occurs before or after the haustorium attaches the host surface [10]. Once the crop is infected, it is difficult to fend off the exhaustion of nutrients created by the attached parasite. Then, chemical control is the most frequent commercial method, albeit its application needs to observe specific recommendations for each parasitic life form [12].

In this review, we describe the current state of knowledge on the mechanisms controlling the infection process of parasitic weeds, and the defense systems and protection strategies employed by their hosts. 

## 2. Infection by Parasitic Weeds

**Figure 2:**
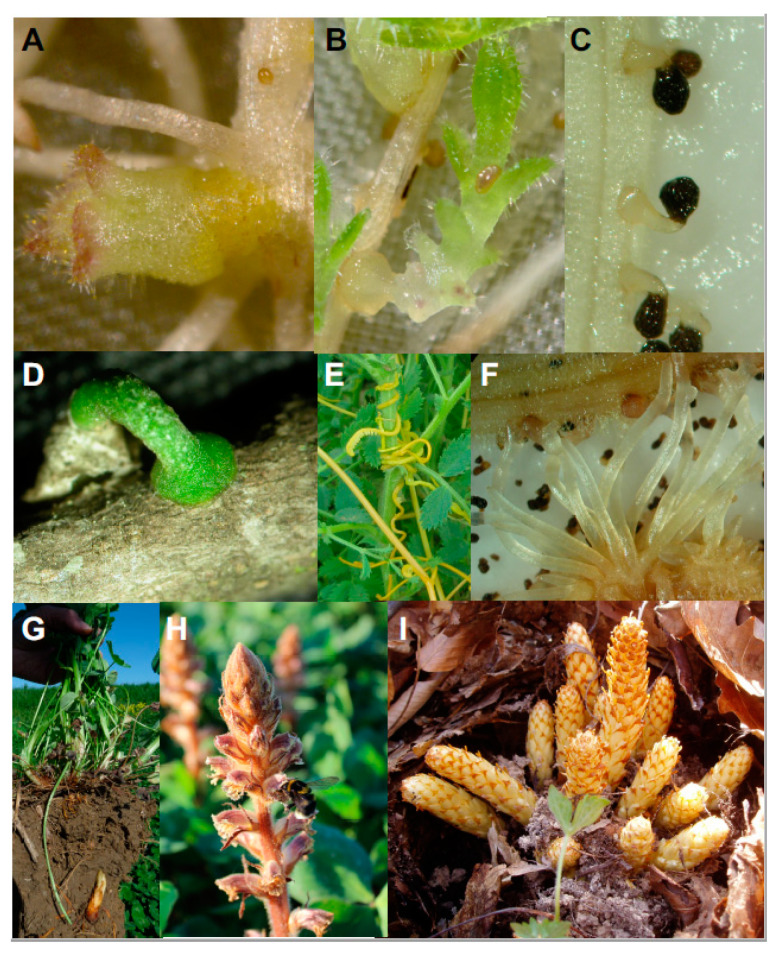
Haustorial penetration of host plants and maturation of parasite. Shown are representative photographs of terminal (**A**–**D**,**G**–**I**) and lateral (**E**,**F**) haustorium penetration and maturation stages. (**A**) Young hemiparasitic weed *Alectra vogelii* infecting the root of cowpea; (**B**) young hemiparasitic weed *Striga hermonthica* infecting the root of sorghum; (**C**) young holoparasitic weed *Phelipanche aegyptiaca* infecting the root of *Medicago*; (**D**) young hemiparasitic weed *Viscum cruciatum* infecting an olive tree stem; (**E**) hemiparasitic weed *Cuscuta campestris* infecting a chickpea stem; (**F**) mature anchorage roots of *Phelipanche aegyptiaca* infecting the root of vetch; (**G**) underground shoot of *Orobanche minor* growing on clover roots towards soil surface; (**H**) emerged shoot of *Orobanche minor*; (**I**) emerged shoots of *Conopholis americana* feeding on oak roots.

All forms of parasitic weeds, facultative or obligate parasites, hemiparasites or holoparasites, root parasites, or stem parasites interact with the host crop by means of the haustorium [7]. Two main types of haustoria exist in root parasitic plants [1,8]. The terminal haustorium is typically developed by obligated parasitic weeds at the tip of their embryonic radicle. The lateral haustorium is developed as an extension in hemiparasitic radicle and mature roots of hemiparasitic and holoparasitic weeds. In aboveground parasitic weeds that infect crop stems and leaves, lateral haustoria develop as lateral extensions of their parasitic stems (Figure 2). The formation of a mature haustorium involves four developmental stages: Haustorium initiation, host invasion, establishment of host-parasite vascular continuity, and the creation of a parasitic sink that will be accepted by the crop as one of its own. Several mechanisms of crop location adapt the timing of haustorium development to the resource availability of its hosts [13].

### 2.1. Crop Location for Germination, Host Trophic Growth and Haustorium Initiation

In contrast to parasitic plants growing in wild ecosystems, where host plant communities provide predictable nutrition to the parasite [14], the supply of resources in agricultural lands for the persistence of parasitic weed communities is affected by changes of cropping systems attending market demands for specific crops, or novel market availability of resistant varieties. Sudden changes in cropping systems impact parasitic weed communities differently, depending on their degree of autotrophy and feeding strategy, being obligated root parasites of annual crops submitted to the higher pressure. To adapt their parasitic life with the timing of crop resource availability, parasitic weeds use mechanisms of host location, which, depending on the degree of autotrophy and feeding strategy, involve up to 3 steps: Host-induced germination, host-tropic growth, and host-induced haustorium initiation [13]. The high pressure to timely detect the host in resource-unpredictable agroecosystems promotes genetic and epigenetic changes, rapidly leading to new weed biotypes with adaptation capabilities to new agricultural practices, climatic areas, new hosts species, and resistant cultivars [15,16].

#### 2.1.1. Germination

The root parasites in the genera *Orobanche/Phelipanche* and *Striga* are obligated parasitic weeds with a high degree in host dependency and host specificity [17]. In absence of host contact, the seedling has a limited growth capacity, allowing it to elongate only a few millimeters before its resources are exhausted, a few days after germination. In agricultural fields, the vulnerability of their seedlings is much higher than in any other weed. To survive, their germination strategy is built on the combination of four seed traits: Seed size, seed number, embryo longevity, and seed dormancy. *Orobanche/Phelipanche* and *Striga* seedlings do not compete for resources in their pre-attached young life stages and therefore the parent plants produce a tremendous number of tiny seeds with high colonization capacity [18]. These seeds penetrate easily into the soil, even in untilled soils, and are easily dispersed by wind, increasing the chances to encounter susceptible hosts in adjacent fields, allowing the population to persist. The seed viability is preserved over time by long embryo longevity and two cooperative processes of dormancy that delay germination until detection of a nearby host. The first dormancy process is a temperature-dependent cyclic dormancy, being non-dormant in the host-growing season and dormant in the next. During the non-dormant season, seed receptors are activated to detect germination stimulants exuded by host roots [19,20,21]. If the chemical signal is not detected, germination will remain inhibited during the non-dormant season and will re-enter the dormant phase the next season. The amount and nature of exudation of germination stimulants by crop roots varies depending on the crop species, the phenological stage, crop nutritional status, and the growing season [19,22,23,24,25,26,27,28,29,30]. For each parasitic weed species, there are multiple seed receptors within a family of α/β-hydrolases that specifically detect the stimulants differentially exuded by their host species [31].

Different feeding styles in other parasitic weeds, like in the host-generalist *Cuscuta* and the facultative parasite *Ramphicarpa*, make the likelihood of their finding nutrients less dependent on finding a specific host right after germination. Consequently, their seeds tend to be larger and are not dependent upon chemical signals for germination, although their seedlings will detect the host through different signals at later growth stages [13]. Their seeds have typical dormancy mechanisms in autotrophic weeds such as physical dormancy induced by a thick seed coat in *Cuscuta*, that preserves seedbank viability against seed predators and allows a staggered germination over time or a physiological dormancy in *Ramphicarpa* that inhibits germination in the dark, allowing the buried part of their seedbanks to delay germination. Once those regular mechanisms of dormancy are broken, their seeds germinate when temperature indicates the growing season for favorable establishment and reproduction [32,33,34]. 

#### 2.1.2. Host-Tropic Growth 

**Figure 3:**
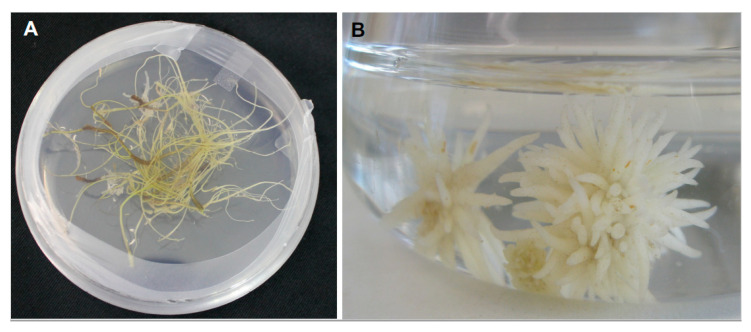
Haustorial competent root cultures of obligated root parasitic weeds. Shown are representative photographs of (**A**) *Striga hermonthica*; (**B**) *Phelipanche aegyptiaca*.

**Figure 4:**
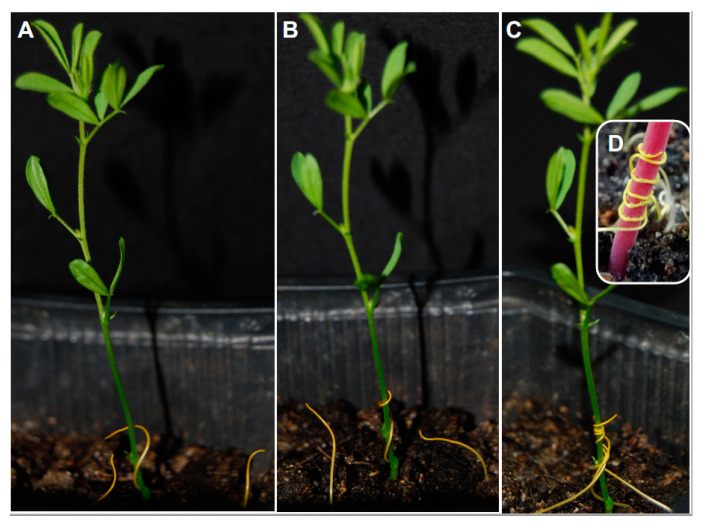
Directional growth of five-day-old *Cuscuta* toward its host. Shown are representative photographs taken at 12-h intervals of the growth of *Cuscuta* and its host lentil. (**A**) *Cuscuta* seedlings rotate in a counterclockwise rotation; (**B**) *Cuscuta* seedling bending guided toward host stem; (**C**) *Cuscuta* coiling and adhesion; (**D**) formation of adhesive discs.

After *Striga* and *Orobanche/Phelipanche* germination, a radicle emerges from the seed and rapidly explores the nearby underground environment to reach the host surface. *Striga* radicle grows by cell growth and apical cell division while *Orobanche/Phelipanche* radicle grows by cell elongation [18]. Although chemotropism has not been demonstrated yet, in vitro observations of the seedling radicle redirecting its growth directly toward the host root have made different authors suggest that host chemi-localization by the radicle could be part of the *Striga* and *Orobanche/Phelipanche* infection process (Figure 2B,C) [13,18,35,36,37,38]. Seeds that germinate a distance longer than 4 mm lose their polarity and exhaust their viability before they can reach the host and therefore such chemotropism would be an advantageous mechanism [38,39]. Although the seedling viability rapidly expires in the soil or in rhizotron experiments, they can be grown on media culture to produce root cultures and regenerate flowering plants [40,41,42,43] (Figure 3). *Cuscuta* seedling viability expires without a host in 3 to 7 weeks depending on the photosynthetic activity of each *Cuscuta* species [44]. *Cuscuta* explores the nearby aerial environment using a rotative movement (Figure 4). Volatile chemicals and far-red light, indicative of proximity of vegetation, guides the *Cuscuta* movement towards the host [45,46,47]. 

#### 2.1.3. Initiation of Haustorium

**Figure 5:**
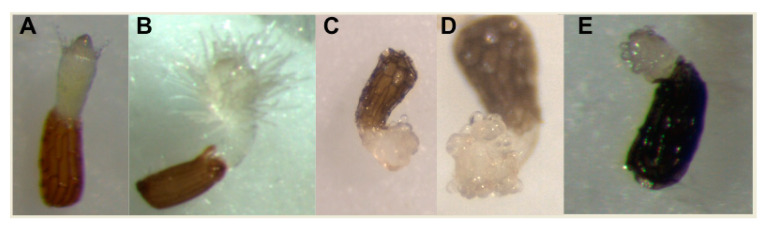
Haustorial hairs and papillae on root parasitic weeds. Shown are representative photographs of haustorial hairs (**A**,**B**) and papillae (**C**–**E**) formed in seedlings of root parasitic weeds. (**A**) *Ramphicarpa fistulosa*; (**B**) *Striga hermonthica*; (**C**,**D**) *Phelipanche ramose*; (**E**) *Orobanche cumana.*

The first stage in haustorium development is initiated upon host detection through chemical and physical signals, which develops an adhesive structure that cements the parasite to the host surface from which the invasive organ subsequently develops [7,48,49,50,51]. Perception of haustorium-inducing signals promotes a cessation of parasite root growth with a rapid swelling. Auxin biosynthesis genes are upregulated at the epidermal cells near the contact site [52]. These cells divide and elongate, becoming, depending on the parasitic species, either haustorial hairs or papillae covered by adhesive secretions to serve as the anchoring device (Figure 5) [53,54,55]. The chemical nature of the adhesive glues is not completely clear but literature indicates that it differs for different parasitic species. For example, the adhesive compounds have been described as hemicelluloses in *Agalinis* [56], pectinaceous mucilaginous material in *Triphysaria* [57], compounds that stain positive for carbohydrate material in *Orobanche/Phelipanche* [53], while in *Striga* these compounds stain negative for carbohydrate material but give a positive result with safranine [58]. In the stem parasite *Cuscuta,* epidermal cells at the parasite-host contact sites dedifferentiate into disk-like meristems forming the pre-haustorium (Figure 4D). Some of these cells become elongated and secrete pectinaceous substances to seal the parasite haustorium to the host surface. Tactile signals, light spectrum, and phytohormones play a pivotal role in the development of the *Cuscuta* pre-haustorium [13,50,59,60,61].

### 2.2. Host Invasion and Establishment of Vascular Connection

Infections by root and shoot parasites modify various cellular processes both in crop and parasitic tissues at the site of crop-parasite contact. Haustorium penetration upregulates genes such as expansins, arabinogalactan proteins, or xyloglucan-endotransglucosylases with a role of rearranging the cell wall [62,63,64]. Parasitic weed gene expression in the haustorium is similar to gene expression in roots of non-parasitic plants [65]. In some root parasites the haustorium initiates from root pericycle as it occurs for lateral roots of no-parasitic plants [66,67]. In other parasitic species the sequence of events initiates in cortical cells [7,68,69] Subsequently, the epidermal cells at the sealed parasite-host root interface become densely protoplasmic and enlarge rapidly, creating a mechanical force to enter the host. The radicle secretes cell wall-modifying enzymes such as pectinolytic enzymes, polygalacturonase, rhamnogalacturonase, and peroxidase [39,70,71]. The combination of mechanical force and enzymatic digestion of host cell walls pushes aside the cells of the host epidermis, cortex, and endodermis from the pathway of weed invasion [53]. Differentiation of vascular elements can occur concomitantly with penetration of endodermis [54] or when the intrusive cells reach the host xylem [72]. Comparative transcriptomics has shown that during haustorium evolution, parasitic plants may have recruited genes from unrelated plant structures with invasive functions (e.g., pollen tubes) and repurposed these genes in the haustorium [65]. Auxin flow is critical for xylem continuity and determines the polarity of the haustorium to allow its function as a root, absorbing water and solutes [73]. In parallel with the formation of xylem-xylem connections, depending on the parasite species, there is the development of sieve elements in haustoria [74]. Some holoparasites such as *Orobanche/Phelipanche* species develop direct symplastic connections through plasmodesmata [75]. The invasive haustorium of *Cuscuta* grows through the cells of the host stem, forming searching hyphae that elongate and reach the host xylem to become xylem cells that establish the crop-parasite bridge [32]. *Cuscuta* haustorium has been compared with a modified adventitious root; however, it develops in the pre-haustorium from dedifferentiation of cortical parenchyma in a different manner that the adventitious roots develop within the cambium [76,77,78]. Additionally, the SHOOT MERISTEMLESS-like factor plays a crucial role in *Cuscuta* haustorium but it is not involved in root development [78].

### 2.3. Maturation of Absorptive Sink

**Figure 6:**
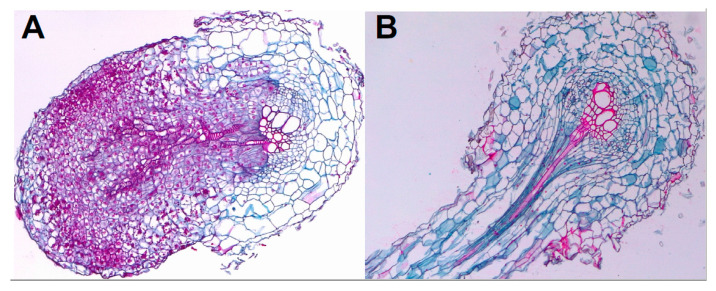
Xylem to xylem junctures in sunflower root. (**A**) Longitudinal section of *Orobanche cumana* seedling connected to sunflower vascular system (shown in transversal section); (**B**) Longitudinal section of sunflower lateral root developed from main root sunflower root (shown in transversal section).

As in lateral roots, the vasculature of a mature haustorium extends from the corresponding host tissues oriented perpendicular to the main axis of host vascular system (Figure 6A). In addition, in the infestation site of some root parasites the host cambium is oriented in line with the cambium of the parasite, therefore perpendicular to the main axis of the host root. The same does not occur for endodermis, cortex, or epidermis, consistent with the fact that the haustorium assimilates nutrients and water from the crop vasculature rather than from the soil [8]. While hemiparasites are by definition photosynthetically active and usually considered parasites mainly for water and minerals, their photosynthesis is not sufficient to fulfill the growth requirements and their haustorium also extracts carbon from the host [79,80]. Holoparasitic weeds like *Orobanche/Phelipanche* and some species of *Cuscuta* do not photosynthesize and have low levels of transpiration and obtain all their carbon, nitrogen, and other minerals from the host mostly from the host phloem [81,82,83]. 

Despite several anatomical and biochemical studies of types of vascular connections and transferred nutrients between host and different parasitic weed species the physiology of parasitic phloem loading in parasitic weeds is not clear [6]. Direct symplastic connections through plasmodesmata [75] and raffinose series oligosaccharides [84,85] seem to indicate the symplastic loading style in holoparasites. In contrast, several indications support the hypothesis of an apoplastic pumping of extracellular sucrose into hemiparasite phloem: (i) The apoplastic loading is most common in herbaceous plants and in many hosts of hemiparasites [86]; (ii) symplastic connections have not been found in hemiparasitic weeds except for *Striga gesnerioides* [87]; and (iii) the apoplastic separation between hemiparasite and the host would reinforce a concentration gradient of solutes [6]. 

The relative sink strength among different plant organs is determined by factors such as the activity of invertase enzymes and sugar transporters, vascular pressure, the density of unloading sites, and the developmental coordination among competing sinks. The nutrient sink created by parasitic weeds gains competitive strength against authentic crop sinks in the host also by means of these same mechanisms. The haustorium cells metabolize the host nutrients into a parasite-specific metabolic profile, creating an osmotic potential favorable for the nutrient flow towards the parasite [84,88,89,90,91]. High levels of potassium, cytokinin, and abscisic acid in the parasite may keep the parasitic stomata open and decrease hydraulic conductivity to promote the host-to-parasite flow relative to other sinks [6,79,92,93,94]. The dense parasitic seedbanks in agricultural lands with germination synchronized with the specific phenology of the crop creates a coordinated attack of several parasites, creating multiple and simultaneous parasitic unloading sites before the crop has the opportunity to redirect the resource allocation towards host reproductive organs [28,95,96]. Breeding crops with coordinated and precocious pod filling relative to the timing of parasitic attachment suppresses the strength of the parasitic sink in susceptible crops [97]. 

## 3. Effect of Parasitic Weed Infection on the Crop

Crop development from germination to reproduction occurs according to a predicted alternation of priorities in sink organs ultimately destined to allocate crop resources toward crop reproduction. Parasitic weed infection strongly reduces crop harvest by either (i) disrupting the crop orchestration of resource allocation, altering dry matter partitioning between crop organs prioritizing those adjacent to the parasite (i.e., host roots in the case of root parasites and host shoots in the case of shoot parasites [83,98]) and subsequently diverting the destination of crop resources into the building of parasite biomass to fulfill the parasitic reproduction, or (ii) inducing pathogenic effects on the crop photosynthetic and nutrient uptake machineries [83,98,99,100]. The extent to which parasitic weeds affect the crop growth, biomass partitioning, and nutrient status differs depending on the differing feeding styles of the parasites [101]. The infection by some hemiparasitic weeds, such as *Striga*, induces crop biomass depression greater than the biomass accumulated by the parasite. *Striga*, like other plant herbivores and pathogens, decreases host productivity by lowering rates of crop photosynthesis [99,102,103]. In contrast, nutrient sinks created by the achlorophyllous *Orobanche cernua* or *Cuscuta reflexa* affect the crop in a fashion similar to mycorrhizal symbionts, increasing rates of net photosynthesis [98,100,104]. *Orobanche*-infected crops are smaller but have an increased rate of carbon acquisition with the end result that infected plants maintain an equivalent resource budget for the combined *Orobanche*-crop-infected biomass complex compared to that of an uninfected plant [100]. 

The parasitic sink strength increases with the density of unloading parasitic attachments up to maximum feeding capacity from which the parasites compete among them and a negative relation between number of parasites attached per plant and weight of individual parasites is observed [96,100,105,106]. For each parasitic species the maximum feeding capacity varies with the crop species they infect and may suggest a trade agreement by which the parasite allows the crop to grow and produce the resources the parasite needs to ensure parasitic reproduction [50,96]. Conserved symbiotic pathways that mediate mechanisms that regulate the extent of colonization by *Rhizobium* and arbuscular mycorrhizal fungi could also be in place in parasitic weeds [107]. The parasitic weed sink strongly competes with other host sinks at the greatest expense of host reproductive organs [95,96]. 

## 4. Phenotypic Expression of Resistance

**Figure 7:**
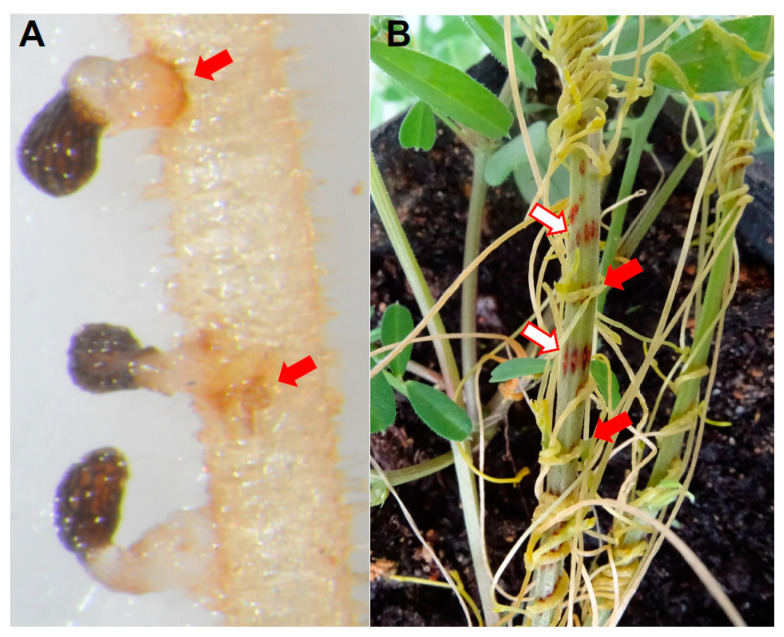
Hypersensitive-like response developed on host in response to parasite attack. Shown are representative photographs of host root responses to attempted penetration by parasite haustorium. (**A**) *Phelipanche aegyptiaca* on *Vicia arthropurpurea* and (**B**) *Cuscuta campestris* on *Vicia sativa*. Red arrows point at hypersensitive-like response at host-parasite interface both in *Phelipanche* and *Cuscuta*. White arrows point at *V. sativa* sites where *Cuscuta* haustorium was manually removed to make the resistance response more visible for explanatory reasons.

Parasitic weeds differ in host range and crop species differ in their susceptibility to parasitic weeds, being resistant to some parasitic species but highly susceptible to the attack of others. Additionally, within host species, some genotypes will be capable of supporting growth and reproduction of a specific parasitic weed species, whereas others will have contrasting levels of resistance and susceptibility [97,108]. Defense-related genes are induced in the parasitized plant during parasitic weed penetration both in susceptible and resistant interactions, indicating that susceptible hosts detect at early stages the penetrating parasitic weed as an alien and not as coordinated growth of an attractive sink [109,110,111]. During the susceptible response, the parasite must circumvent the first response to later become a compatible sink. However, some wild species, landraces, and a few cultivars have innate or inducible resistance mechanisms to attack by *Striga*, *Orobanche/Phelipanche*, or *Cuscuta* (Figure 7) [10,38,112]. Post-attachment resistance operates at several levels. These processes initiate when the parasite haustorium attaches to the host surface and attempts to penetrate host tissues to connect with the vascular system. Among the processes described are: (i) Abiosis, the synthesis and release of cytotoxic compounds such as phenolic acids and phytoalexins; (ii) the rapid formation of physical barriers to prevent possible pathogen ingress and growth (e.g., lignification and other forms of cell wall modification at the host-parasite interface); (iii) the release of reactive oxygen species and activation of programmed cell death in the form of a hypersensitive response at the point of parasite attachment to limit parasite development and retard its penetration; and (iv) prevention of the parasite establishing the essential functional vascular continuity (i.e., xylem-to-xylem and/or phloem-to-phloem connections) with the host, delaying parasite growth followed by parasite developmental arrest and eventual death [113].

## 5. Evolution of Host Specificities and Races 

Some *Orobanche/Phelipanche* species are major phytopathogens on several important crops in throughout North Africa, the Middle East, and Southern and Eastern Europe, and their footprint continues to expand on these crops as well as their host range continues to evolve. Indeed, recent reports reveal that non-weedy *Orobanche/Phelipanche* species can also infest crop species as an ecological opportunity arises. For instance, the infestation of celery (*Apium graveolens* L.) by *Orobanche nana*, known as a non-weedy parasite, was reported for the first time in Italy in 2014 [114]. Several other reports indicated the infestation of new compatible targets by weedy *Orobanche/Phelipanche* species when the density of this new but unfavored hosts increase [115,116,117,118,119,120].

The eco-evolutionary dynamics that cause parasitic plants to infest new hosts can be of two types: (i) An extension of the range of their natural hosts or (ii) a host specialization occurring as a result of genetic modifications impacting a major key step of the parasitic process (i.e., detection of germination stimulants or haustorium-inducing factors). In *Phelipanche ramosa*, it appears that host specialization is acting, rather than an extension of the host range [18]. This parasitic species is native to Europe and Western Asia and has been introduced to most continents. It is known for its broad host range as it can parasitize more than 50 different hosts including crops and vegetable [121]. However, at least three genetic groups of *P. ramosa* have been identified in France, differentiated in particular by their host preferences (oilseed rape, hemp, and tobacco, respectively) [122,123]. These different pathovars of *P. ramosa* are distinguished by their differential response to germination stimulants and by a higher fitness (i.e., greater reproductive success) on some crop hosts than on others [123,124,125]. Host specialization has also been observed in *Orobanche cumana*, which in contrast with *P. ramosa* exhibits a narrow host range. *O. cumana* is the most serious constraint for sunflower cultivation in Southern and Eastern Europe and in North Asia. The wild ancestor of *O. cumana* is found in Caucasus, Southern Russia, and on the Black Sea coast where isolated populations parasitized *Artemisia* spp. The first observation of *O. cumana* parasitizing sunflower was made in Russia in 1890. At the end of the 19th and beginning of the 20th century, the attacks of *O. cumana* in Russia were so severe that it threatened the production of sunflower oil. Breeders have therefore developed sunflower varieties or hybrids resistant to the forms of *O. cumana*, called races, which have proved to be increasingly aggressive because of their ability to bypass the successively introgressed resistant genes. To date, at least 7 races (A–G) have emerged along with seven resistant genes (*Or1*–*Or7*). The same type of “arms race” could be observed in both species of witchweeds, *S. asiatica* [126] and *S. gesnerioides* [127].

While significant progress has been made in understanding the biology behind the development of parasitic plants and their parasitic strategies, the biology underlying their adaptation to a host remains unknown. Host specialization probably involves genetic mutations. However, several lines of evidence, including rapid host adaptation and minimal or no genetic differentiation between pathovars and races of *Orobanche/Phelipanche* and *Striga* species, indicate that host changes could also involve epigenetic modifications. One major goal would be now to determine how host preferences arise and to evaluate the differential contributions of genetic change and epigenetic priming mechanisms for host specificity in parasitic weeds.

## 6. Resistance Genes to Parasitic Plants

The identification of differentially resistant genetic variants within host germplasms and construction of populations segregating for the resistance phenotype permitted researchers to identify individual genes or groups of genes conferring resistance [10,128,129,130]. In most cases, resistance to *Striga spp*. in the grasses (sorghum, millet, rice) appears to be polygenic with both major and minor genes with a large genotype by environment interaction [10]. Resistance often appears to involve several mechanisms, is often weak, and tends to break down in the presence of new geographic or physiologically specialized forms of the parasite. Resistance to *S. gesnerioides*, which primarily attacks dicots, appears to be mainly monogenic [131], and in cowpea where it has been extensively studied, resistance appears to be race-specific with multiple pathotypes of *S. gesnerioides* and multiple resistance genes in the cowpea genome [132]. The host differential response in cowpea cultivars allowed the construction of genetic populations segregating for the race-specific phenotype, and subsequently the use of a marker-assisted positional cloning strategy by [133] to isolate a dominant gene from cowpea termed *RSG3-301*, conferring resistance to *S. gesnerioides* race SG3. *RSG3-301* encodes a typical nucleotide-binding domain and leucine-rich repeat containing (NLR) protein with a N-terminal coiled-coil domain (CC), followed by a central nucleotide binding site (NBS) and a C-terminal leucine-rich repeat (LRR) domain. 

The characterization of *RSG3-301* led Li and Timko [133] to suggest that race-specific *Striga* resistance in cowpea is an example of effector-triggered immunity (ETI) in which intracellular NLR proteins (such as RSG3-301) are activated either directly or indirectly upon recognition of pathogen/parasite effectors [134,135]. 

Membrane-bound receptors in tomato (CuRe) and sunflower (*HaOr7*) functioning as pattern recognition receptors (PRRs) were discovered [136,137] that appear to be involved in pathogen-associated molecular patterns (PAMP)-triggered immunity, the first level of plant defense responses [138,139]. Subsequently, Jhu et al. [140] identified a resistance gene in tomato dubbed CuRLR1 that appears to also be involved in ETI against *Cuscuta*. Most of the resistance genes to *Orobanche* have been discovered and used for sunflower breeding programs for *O. cumana* resistance [141]. Qualitative resistance to *O. cumana* is controlled by major genes and is race-specific. Thus, seven dominant resistance genes *Or1* to *Or7* have been described corresponding to 6 races of *O. cumana* A to F. A more virulent race G exists for which no resistance has been characterized to date. However, among these resistance genes only the *Or5* and *Or7* genes have been previously mapped. *Or5* confers resistance to the race E and has been shown to interact with the *AvrOr5* avirulence gene agreeing with the gene-for-gene hypothesis in the *O. cumana*-sunflower pathosystem [142,143]. Recently, Duriez et al. [137] deciphered the function of *Or7* conferring resistance to *O. cumana* race F by preventing the connection of the parasite to the vascular system of the sunflower roots. *Or7* encodes a membrane receptor-type LRR kinase, suggesting the existence of a parasitic AVROR7 avirulence protein. In *P. ramosa*, despite the identification of resistances in winter oilseed rape [144], nothing is known about the underlying molecular processes.

## 7. Parasite Effectors

The deployment of secreted effectors to block host resistance responses is observed in multiple host-pathogen interactions including pathogenic microbes, fungi, and nematodes [145,146,147]. Recently, Su et al. [148] used transcriptomic profiling and transgenic expression analysis to identify a novel secreted effector protein from *S. gesnerioides* termed SHR4z, which blocks host plant immunity in the multi-race resistance cowpea B301. SHR4z suppresses the hypersensitive response (HR) triggered by *S. gesnerioides* race SG4 by interfering with signal transduction pathway leading to HR activation upon parasite attack. The exact mechanism remains to be determined, but SHR4z is structurally similar within the N-terminal region to the SERK (somatic embryogenesis receptor-like kinase) subfamily of LRR-RLKs and is predicted to interact with the BTB/POZ interface of VuPOB1, a PUB E3 ligase family member that appears to be a positive regulator of HR in cowpea. 

A set of five candidate effector proteins that suppress known plant defense pathways in Arabidopsis including two LRR SERK-like proteins similar to SHR4z have also been reported by Clarke et al. [149]. Additional characterization of the common aspects of signal transduction pathways involved in response to parasitic weeds is clearly needed. 

## 8. Transcriptome and Proteome Analysis of Resistance 

The immune response of host plants to parasitic plants is also likely multi-faceted. Similar suites of genes are observed to be upregulated during resistance responses to *Orobanche* and *Striga* and it is becoming increasingly clear that the SA signaling pathway and to a lesser extent the JA signaling pathway play important roles in the activation of resistance to parasitic plants. In other plant-plant pathogen interactions the SA and JA defense pathways can interact either antagonistically or synergistically [150]. The SA pathway is often activated in response to biotrophic fungal pathogens, leading to the expression of suites of PR genes, whereas the JA pathway is often important in resistance to necrotrophic pathogens and insect pests. A number of studies have evaluated the effectiveness of SA applications to hosts to prevent parasitism by *Orobanche* spp. SA consistently promoted resistance, in the case of clover roots infected with *O. minor*, by the activation of defense responses leading to lignification of the endodermis [151]. SA analogues have also been proved to induce resistance in oilseed rape against *Phelipanche ramosa* [152]. To date, SA-inducing chemicals have not been widely applied to field-grown crops to test their effectiveness as part of a control strategy. The induction of genes involved in JA biosynthesis has been observed in compatible interactions between *Orobanche* species and their hosts [153] but the involvement of JA in resistance is less clear [151]. A decade ago, in a study of resistance in tomato to the shoot parasite *Cuscuta pentagona*, a role for both JA and SA was also proposed [154]. The importance of several genes for *A. thaliana* resistance and susceptibility to *P. aegyptiaca* was examined by Clarke et al. [149] who found that functional crosstalk between SA and JA signaling is required to support the attachment and development of the parasite and that the parasite specifically manipulates these pathways to ensure success. Manipulation of the crosstalk can specifically limit the ability of *P. aegyptiaca* to parasitize *A. thaliana*. In particular, several host genes involved in JA and SA biosynthesis and signaling are required for full host susceptibility and the putative immunity hub protein PFD6 is a critical component of the host immune response that limits the progression of *P. aegyptiaca* parasitism.

## 9. Strategies for Effective Parasitic Weed Management

Due to the high fertility rate of parasitic weeds, their management must inhibit not only the loss of crop yield but also parasitic flowering. A given control method could not be considered completely successful if it does not inhibit parasitic weed seed bank replenishing at least by 95% [155,156,157]. Commercial strategies for parasitic weed are currently based in crop resistance and chemical control. The processes of host-induced parasitic germination and haustorium formation are the most frequent targets for the development of resistant cultivars [112,158,159,160]. Sources of resistance against these mechanisms are scarce among crop germplasm collections being more frequent in wild relatives to crop species [10,11]. The success of the slow and difficult process of traditional breeding methods that adds one resistance gene at a time is quickly overcome by parasitic weeds due to: (i) The existence of individuals among the dense and heterogeneous seed banks capable of overcoming the resistance of the new cultivar before its first cultivation and (ii) after the cultivation of the resistant cultivar, the capacity of stressed parasitic weeds to mutate, evolving virulence-enhanced parasitic biotypes much quicker than the breeders’ capacity to develop the resistant cultivar [141,161]. 

The use of herbicides as a strategy for parasitic weed control [12] is mainly determined by: (i) Whether the parasite and the crop are attached, the herbicide is translocatable, and if the herbicide is selective enough to kill the parasite without damaging the crop; (ii) if the parasite is holoparasitic and achlorophyllous since photosynthesis-inhibiting herbicides cannot be used for their control [157,162,163], and (iii) in the case of root parasitic weeds, the main crop damage is done during the underground parasitic life stages and therefore post-emergence herbicides do not prevent yield losses [164,165]. The close interaction of parasitic weeds with the host crop allows the use of systemic herbicides to control young parasites before they provoke the crop damage [166]. The systemic herbicide is applied to the crop foliage and delivered to the shoot or root parasites either via the haustorium or through exudation to the rhizosphere from the crop roots [167,168,169]. The systemic herbicides used for parasitic weeds include inhibitors of aromatic (glyphosate) or branched-chain amino acid synthesis (imidazolinones and sulfonylureas), inhibitors of vitamin folic acid (asulam), inhibitors of glutamine synthetase (glufosinate), or hormonal herbicides (2,4-D and dicamba) [169,170,171,172,173]. The efficacy of each herbicide varies with the parasitic weed species. For example, glyphosate and imazamox have herbicidal effects on root parasitic weeds *Orobanche*/*Phelipanche*, but do not kill *Cuscuta* seedlings; whereas *Cuscuta* are very sensitive to glufosinate [169]. In addition, the intrinsic killing effect of a given herbicide towards a specific weed genotype can be ameliorated by the quality of interaction between the host and parasite, through the quality of haustorium connection, or nutritious capacity of host genotypes [169]. Therefore, the use of herbicides as a strategy must be specifically crafted depending on which parasite-crop species combination is being targeted and on the availability of information on the specific herbicide and herbicidal doses that are sublethal for the crop but can be delivered in a lethal doses to the parasite, and the availability of crop varieties with herbicide resistance [157,166,174]. The success of herbicidal treatment for the control of parasitic weeds can be high, but its sustainability is compromised by the same general problems observed in the deployment of herbicides in general for weed control, namely (i) the predicted emergence of weed resistance to herbicides [175]; (ii) the lack of development of new herbicide mechanisms of action to counteract the emergence of resistance [176]; and (iii) unwanted non-target effects either disrupting biodiversity due to the low herbicide specificity [177,178] or compromising crop immunity against other pests [179,180]. 

Biotechnology has the potential to eliminate the bottleneck in parasitic weed management created by the scarcity in both herbicide mode of actions and resistance sources. As such, it could create opportunities to obtain control strategies based in biologically derived alternative chemistries, to protect the sustainability of existing resistant mechanisms by creating resistance-enhanced crops through gene pyramiding, and to expand the available number of resistant genes for breeding beyond the limited gene pool of a given crop and its wild interbreeding relatives [157,163,181]. Besides the above-mentioned control methods, other non-chemical strategies such as soil fertility amendments or solarization have proven effective in some cases of small-holder farmers or high-value specialty crops [2,12,164,182,183,184].

### 9.1. Bioherbicides

Given the low availability of herbicides against parasitic weeds and the prediction that parasitic weeds will evolve resistance to herbicides [175], the sustainability of chemical control depends on the discovery of new herbicides. The special biology of parasitic weeds limits the number of metabolic pathways that can be targeted by current commercial herbicides but at the same time generates opportunities for the discovery of parasitic weed-specific herbicide targets. For example, biologically-derived chemicals with activity of suicidal induction on essential life stages such as germination or haustorium formation away from the host have a specific killing effect in the parasitic weed while potentially minimizing unwanted health effects on non-target organisms and environment [185,186,187]. Biologically-based weed control methods will play a role on the future of weed management, because of their relatively low cost, long-term sustainability, and environmental friendliness [188]. According to the terminology of the U.S. Environmental Protection Agency, the following types of control strategies can be considered bioherbicides: (i) Microbial herbicides, (ii) biochemical herbicides, and (iii) allelopathic plants that secrete herbicidal compounds [188]. 

Microbial herbicides are plant-pathogenic or non-pathogenic microbes living or not, mixed in or not with their metabolites [188]. The numerous microbes studied for biological control of parasitic weeds have been reviewed by Watson [189]. There are many articles studying, under controlled conditions, the biological control of parasitic weeds by microbes, but few describing an adequate amount of control for farmers. Few biocontrol agents meet the conditions of sufficient virulence, host specificity, and ease of production and application for field use. These limitations could be overcome by biotechnological approaches of virulence enhancement. Mutants and transgenic variants of natural pathogenic isolates of parasitic weeds showed improved control levels through overproducing and secreting either toxic compounds or inductors of suicidal germination [181,190,191,192]. Biochemical herbicides are compounds of microbial or plant origin. Research with toxins from microbial and plant origin and certain naturally occurring chemicals has demonstrated that additional herbicides may be developed targeting the pre-attached parasitic seedlings and therefore inhibiting the haustorium formation on the host surface [49,193]. Recent developments of high-throughput screenings of metabolites on seeds and seedlings of parasitic weeds will speed up herbicide discovery efforts [194]. Allelopathic crops delivering in situ herbicidal action against parasitic weeds in agroecosystems should also be considered in future sustainable weed management [195]. The strategy of genetically engineering crops to overproduce and secrete either toxic chemicals or inductors of suicidal germination into the rhizosphere [157,196] is discussed in the section below. Finally, the use of RNA to silence essential weed genes through the process of RNA interference has great potential for weed management. Applied as a spray, RNAi can efficiently kill weeds with sufficient host specificity because sequences can be designed to selectively target a specific weed species [188]. For its application to parasitic weed control, genetically modified crops expressing RNAi (RNA interference) constructs or microRNAs (miRNAs) could also be considered bioherbicides (see below). 

### 9.2. Gene Pyramiding

The search for new sources of resistance has always been a major process in breeding programs in order to generate new varieties resistant to pests. Vertical resistances are mainly governed by major genes that have a gene-for-gene relationship with a pathogen avirulence factor, thus generating a hypersensitive response (HR) to a specific race and most often preventing the development of the pathogen. Although their use is fairly simple, the history of *O. cumana* parasitizing sunflower specifically is remarkable [197]. Indeed, this example has shown that the resistances thus successively generated are unfortunately not sustainable. The other approach concerns the use of horizontal resistances which are regulated by several genes and which have the advantage of being non-specific (all races of parasitic plant weed are then affected) and durable. However, their use in breeding programs is too complex to implement. An alternative would therefore be to pyramid the genes involved in vertical resistance in order to mimic a kind of horizontal resistance. In *O. cumana*, it would therefore be interesting to accumulate resistance genes already identified such as *HaDef1* encoding a defensin [198,199] or *HaOr7* encoding a receptor-like LRR kinase protein and conferring resistance to race F [137]. Similarly, it would be relevant to revisit resistance alleles already characterized (*Or1* to *Or6*) but whose underlying resistance gene remain to be identified [142]. In this way, the generated gene-pyramid sunflower lines could benefit from a potential synergistic effect among those genes, leading to activation of additional biological process. This could lead to increased resistance and prolonged durability of its effectiveness because the parasitic weed would need to overcome a larger number of sunflower genetic pathways to infect. This technology has proven effective to increase resistance to other plant pathogens [200,201,202] but has not been developed against parasitic weeds. 

### 9.3. Genetic Engineering

There have been several attempts to use plant genetic engineering for improved parasitic weed control by introducing resistance to commercial herbicides into crop plants. The types of selectivity mode in herbicide-resistant crops can be metabolic resistance or target site resistance. In theory, crops with metabolic resistance that catabolize the herbicide in inactive forms could only be used in cases when the herbicide reaches the parasite directly, such as in the strategy of crop seed dressing where the herbicide reaches the parasite through the soil or during application of herbicides on crop canopy infected by stem parasitic weeds. However, *Cuscuta* survives the application of glyphosate and glufosinate to glyphosate-resistant and glufosinate-resistant crops [32,169,203,204]. In the study by Nadler-Hassar et al. [169] the authors determined that in the case of glufosinate applications on the canopy of *Cuscuta*-infected glufosinate-resistant oilseed rape carrying the *bar* gene, which encodes an enzyme that acetylates glufosinate to an inactive form, the herbicide had a reduced effect on *Cuscuta* even when unattached *Cuscuta* plants are very sensitive to glufosinate. These authors suggested that although attached *Cuscuta* plants were also sprayed with glufosinate, the amount of herbicide directly absorbed by *Cuscuta* could not have been sufficient to kill the parasite, indicating that in this case the herbicide primarily gets to the parasite through the host and not by direct application. Similarly, *Cuscuta* survives glufosinate treatment attached to LibertyLink^®^ crops [203]. LibertyLink^®^ crops resist glufosinate applications by transgenically expressing phosphinothricin acetyl transferase (PAT) that detoxifies the herbicide [205]. The work by Jiang et al. [206] determined that dodder acquires glufosinate tolerance from the LibertyLink^®^ host through trafficking and inter-specific function of PAT. Engineered crops with target-site resistance are more appropriate for parasitic weeds because the herbicide is applied on the crop canopy and delivered in active form to the root- or stem-attached parasite through translocation. Transgenic tobacco engineered with a mutant form of acetolactate synthase (AS) conferring resistance to chlorsulfuron could be used as an effective means for controlling *Orobanche/Phelipanche* [207]. Similarly, transgenic carrots engineered for imazapyr resistance is effective in controlling *P. aegyptiaca* [208]. Glyphosate resistance conferred expression of mutant 5-enolpyruvylshikimate-3-phosphate synthase (EPSPS) appears to be readily translocated into developing *Phelipanche* and *Orobanche* spp. tubercles, thereby blocking their development [209,210]. 

Apart from herbicide resistance, among the earliest reports of successful control of parasitic weeds through genetic engineering was engineering the resistance against *Phelipanche* spp. with overexpression of the antibacterial peptide sarcotoxin IA [196,211]. The use of RNA interference was shown as a potential strategy for controlling plant parasitism by [212]. These investigators showed that target gene expression in *Triphysaria versicolor* could be controlled by expressing hpRNA against the gene in the host plant since the hpRNA could be effectively translocated across the haustorium junctions. They also showed that reciprocal movement of silencing RNA could move from parasite to host. In contrast, similar attempts to use RNA interference to block *S. asiatica* parasitism of maize engineered with 13 dsRNA constructs against five key parasite metabolic enzymes showed no significant effects on *Striga* [213]. Expression of multiple RNAi gene constructs (e.g., ACS, M6PR, and Prx1) targeting genes in *P. aegyptiaca* resulted in impairment of parasite growth on the engineered hosts [214]. As for herbicide efficacy, the reason for the difference among parasites in response RNAi expression in host plants could be due to the nature of the vascular connections formed in the different parasite interactions. Among those examined, *Striga*-host interactions do not exhibit inter-phloem connectivity that could limit the capacity to exchange small RNA molecules easily. Similar to root parasites, cross-species RNAi can be used to inhibit infection by stem parasites. *SHOOT MERISTEMLESS-like* RNA interference transgenic tobacco results in defects in establishment of *Cuscuta* haustorium [78]. To date, few reports have appeared describing gene editing as a means of controlling parasitic weeds. Those that have appeared have targeted pre-attachment resistance. For example, Butt et al. [215] used CRISPR/Cas9 to disrupt the *CCD7* gene in rice (*Oryza sativa*). The CCD7 knockout plants showed a significant reduction in the stimulation of *Striga* germination, but also exhibited an increase in tillering and reduced height. Bari et al. [216] have recently reported that CRISPR/Cas9-mediated mutagenesis of the *CCD8* gene in tomato resulted in less root stimulation activity of parasitic germination due to loss of root strigolactone, the main parasitic weed germination stimulant exuded by crops, and a significant reduction in parasite infestation compared to wild type tomato plants. Unfortunately, there were negative agronomic effects as a result of the gene editing, including dwarfing, excessive shoot branching, and adventitious root formation.

## 10. Conclusions

As their significance as a constraint to crop productivity worldwide has expanded, there has been an increased interest in the biology of parasitic weeds. The most notable advancements that have occurred in recent years is our better understanding the underlying chemical, molecular, and genetic factors that control pre- and post-attachment interactions between the parasite and its potential host plant; in particular, the exchange of small molecules, protein effectors, and small regulatory RNAs between parasite and host that define compatibility or incompatibility. This has certainly enlightened our appreciation of the complexity of these unique plant-plant interactions and the subtleties of what constitute success or failure during the process or parasitism. To date many similarities have emerged between the molecular mechanisms mediating parasitic plant-host interactions and other pathogen-plant interactions, including the elicitation of host innate immunity. While the intricacies of the interaction between parasitic weeds and their host plants remains in the early stages of discovery, relative to our understanding of other plant-pathogen interactions (e.g., bacterial, fungal, nematode-elicited pathologies), the availability and application of omics-scale data and related functional genomic tools have accelerated research progress in recent years, and pointed to new directions of research. While we have a much greater appreciation of the role of strigolactones and various haustorial initiation factors in the control of pre-attachment interactions, our knowledge of the factors and signaling processes governing post-attachment interactions is still in its infancy. There are still many open questions about exactly how avirulence and effector molecules move from parasite to host, how metabolic or other forms of feedback from the host conditions parasite development, and how input from the parasite modifies host processes and metabolism to make a favorable pairing to satisfy parasite growth and developmental needs. There is an urgent need for better methods and tools for culturing parasites free and in combinations with their host and for manipulating these associations to better dissect the fine points of successful and unsuccessful interactions. Given our current knowledge, the study of parasitic weeds offers exciting opportunities for young researchers, and will likely provide the plant biology community with new insights into the evolution of these plants. More importantly, the hope is that with increased knowledge about host-parasite interactions there will be greater opportunity and success in developing strategies to limit their spread and alleviate their damage to crops worldwide, thus promoting better food security for future generations.
